# Internet addiction associated with right pars opercularis in females

**DOI:** 10.1556/2006.7.2018.135

**Published:** 2019-01-20

**Authors:** Orsolya Inhóf, András N. Zsidó, Gábor Perlaki, Gergely Orsi, Beatrix Lábadi, Norbert Kovács, Anna Szente, Tamás Dóczi, József Janszky, Gergely Darnai

**Affiliations:** 1 Institute of Psychology, University of Pécs, Pécs, Hungary; 2 MTA-PTE Clinical Neuroscience MR Research Group, Pécs, Hungary; 3 Pécs Diagnostic Centre, Pécs, Hungary; 4 Department of Neurosurgery, University of Pécs Medical School, Pécs, Hungary; 5 Department of Neurology, University of Pécs Medical School, Pécs, Hungary

**Keywords:** Internet addiction, pars opercularis, volumetry, gender differences

## Abstract

**Background and aims:**

Structural differences in higher-order brain areas are common features of behavioral addictions, including Internet addiction (IA) as well. Taking into consideration the limited number of studies and methods used in previous studies on IA, our aim was to investigate the correlates of IA and the morphometry of the frontal lobes.

**Methods:**

To observe these relationships, the high-resolution T1-weighted MR images of 144 healthy, Caucasian, university students were analyzed with volumetry and voxel-based morphometry. The Problematic Internet Use Questionnaire (PIUQ) was used to assess IA.

**Results:**

We found significant correlations between PIUQ subscales and the volume of the right pars opercularis volume and gray matter mass in women.

**Discussion and conclusion:**

The increased gray matter measures of this structure might be explained with the extended effort to control for the impulsive behavior in addiction, and with the increased number of social interactions via the Internet.

## Introduction

Internet addiction (IA) is considered as a behavioral addiction disorder with high prevalence (Cheng & Li, 2014). It includes not only the increased use of the Internet, but according to the most popular models of IA, it is characterized by preoccupation, negative mood management, withdrawal, craving, loss of control and interest, and other social and occupational problems (Kuss, Griffiths, Karila, & Billieux, 2014; Van Rooij & Prause, 2014).

IA has two different forms: specific and general IA (Montag et al., 2015). In specific, IA forms the problematic use is confined to a defined activity, like online gaming. In general, IA the behavioral addiction manifests in different activity forms. The relationship between them is strong; they have several common features, for example, consequences and underlying etiologies (Koo & Kwon, 2014). However, these types can also be distinguished. The Internet Gaming Disorder is included in the fifth edition of the *Diagnostic and Statistical Manual of Mental Disorders* (DSM-5; Király, Griffiths, & Demetrovics, 2015; Griffiths et al., 2016) and 11th revision of the International Classification of Diseases (Rumpf et al., 2018). In contrast, general IA is still not mentioned in these nosological classification systems. This has clinical importance, and the reason behind this might be the lack of reliable researches related to general IA.

Similarly to other addiction forms, people with IA continue an activity even if it has clear negative consequences. This behavior is the result of low inhibitory control paired with high impulsivity, deficit in self-regulation, goal-directed behavior, decision-making, and executive functions (Billieux & Van der Linden, 2012). These functions are controlled by the frontal lobe brain regions (Crews & Boettiger, 2009); thus, similarly to other addictions, structural differences in IA could be located in these areas. Earlier studies revealed decreased cortical thickness in the lateral orbitrofontal (Hong et al., 2013; Yuan et al., 2013) and precentral gyri (Yuan et al., 2013). Reduced gray matter volume was found in the bilateral dorsolateral prefrontal and orbitofrontal cortices (Yuan et al., 2011), as well as in the right frontal pole (Kühn & Gallinat, 2015). Although the Internet usage pattern is different between the sexes, gender differences were not examined in the above studies. Females are more likely to use the Internet to communicate (Kimbrough, Guadagno, Muscanell, & Dill, 2013), while online gaming is more prevalent among males (Ko, Yen, Chen, Chen, & Yen, 2005).

With the growing prevalence of the IA, there is an increasing need for comprehensive research. The aim of this study was to measure the relationship between IA and the morphometric features of frontal cortical areas, focusing on gender differences. To obtain more reliable results, we combined two different morphometric techniques [voxel-based morphometry (VBM) and volumetry]. We hypothesized a correlation between IA and the size of frontal areas. Previous studies showed significant differences in Internet usage between genders; therefore, we hypothesized that the connection between the two variables will be different as well. Since women primarily use the Internet for communication, our suggestion is that the increased usage correlates positively with the areas responsible for lingual and social interactions.

## Methods

### Participants

We measured 144 healthy, right-handed, Caucasian adults (64 males; mean age: 22.5 + 2.2, range: 18–30 years), with normal body mass index. All participants used the Internet on a daily basis. None of them reached the clinical cut-off of Beck Depression Inventory (BDI-I; Beck & Beamesderfer, 1974) or State-Trait Anxiety Inventory (STAI; Spielberger, Gorsuch, & Lushene, 1970). The cut-off point was set at 30 of the BDI-I (Beck, Steer, & Carbin, 1988) and at 39 of the STAI (Julian, 2011). None reported alcohol or other substance addiction. Participants were recruited via announcements on university boards, and via online surfaces of the university.

### Measures

#### Questionnaires

We used the Hungarian version of Problematic Internet Use Questionnaire (PIUQ), which has good reliability and validity characteristics (Demetrovics, Szeredi, & Rózsa, 2008). PIUQ consists of three factors: Obsession, Neglect, and Control Disorder.

Obsession subscale contains items regarding obsessive thinking about the Internet and items about withdrawal symptoms (depression and worry) caused by the lack of Internet use. The items of Neglect subscale refer to neglecting everyday activities or social life due to Internet use. The Control Disorder subscale measures the disability to control the Internet use (Demetrovics et al., 2008). The questionnaire contains 18 items, 6 in each subscales. Participants had to rate the items using a 5-point Likert-type scale. According to Koronczai et al. (2011), the suggested cut-off point of PIUQ is 41. Based on this recommended score, 31 subjects for the study population have IA. But in the lack of well-established diagnostic criteria (Kuss & Lopez-Fernandez, 2016; Poli, 2017), we decided to use a multidimensional continuous measure of IA.

During the selection of the participants, we used Edinburgh Handedness Inventory to assess handedness (Oldfield, 1971). According to the Edinburg Handedness Inventory, a handedness index score was computed by dividing the difference between right and left responses by the total number of responses and multiplying the ratio by 100. The scores of our sample ranged from 12.5 to 100 [mean (*SD*) = 73.41 (23.47)].

#### MRI examination

For MRI measurements, we used a 3 Tesla MR scanner (MAGNETOM Trio a Tim System, Siemens AG, Erlangen, Germany) with a 12-channel head coil. For the morphometric analyses, isotopic 3D T1-weighted magnetization-prepared rapid gradient echo images were used: TR/TI/TE = 2,530/11.00/3.37 ms, slice thickness = 1 mm, number of sagittal slices = 176, flip angle = 7°, receiver bandwidth = 200 Hz/pixel, FoV = 256 × 256 mm^2^, matrix size = 256 × 256.

#### MRI data evaluation

##### Volumetric analysis.

Freesurfer v6.0 (http://surfer.nmr.mgh.harvard.edu/) was used for cortical reconstruction and volumetric segmentation of the images. This software provides one of the most reliable automated brain segmentation methods for cortical and subcortical structures. It allows to assess the volume and other morphological features of predefined brain structures (Fischl, 2012). Freesurfer’s semi-automatic anatomical processing scripts (autorecon 1, 2, and 3) were executed on all subjects’ data. Visual verifications were performed for all subjects and error corrections were applied whenever it was indicated. Volume information was acquired according to the Desikan–Killiany–Tourville labeling protocol (Klein & Tourville, 2012). The following bilateral cortical regions were under our interest: caudal and rostal middle frontal gyri, lateral and medial orbitofrontal gyri, pars opercularis (POp), pars orbitalis, pars triangularis (PTr), and precentral and superior frontal gyri.

#### VBM

We used FSL-VBM (http://www.fmrib.ox.ac.uk/fsl) to assess the gray matter volume of the frontal cortex. We carried out an “optimized” VBM protocol with the use of FSL tools (Smith et al., 2004). After brain extraction of the T1-weighted images using BET, we carried out tissue-type segmentation using FMRIB’s Automated Segmentation Tool. Next, we aligned the resulting gray matter partial volume images to the MNI152 standard space using non-linear registration. The resulting images were averaged together with their respective mirror images to create a left–right symmetric study-specific gray matter template. The gray matter partial volume images of all subjects were non-linearly registered to this study-specific template. Modulation of the registered partial volume images followed (to correct for local expansion or contraction owing to the non-linear components of the registration) by multiplying by the Jacobian of the warp field. Then, the modulated segmented images were smoothed with an isotropic Gaussian kernel with a sigma of 3 mm. Finally, to confirm the results of volumetric analysis, we applied voxelwise general linear models on brain structures, which were associated significantly with PIUQ subscales, using permutation-based non-parametric testing (5,000 permutations), correcting for multiple comparisons across space. The results were considered significant for *p* < .05, corrected for multiple comparisons using threshold-free cluster enhancement, which avoids making an arbitrary choice of the cluster-forming threshold, while preserving the sensitivity benefits of clusterwise correction. The whole frontal cortex defined based on the MNI structural atlas thresholded at 0% was selected as region of interest (ROI).

### Statistical analyses

Statistical analysis for volumetry was performed using the SPSS statistical software version 22.0 (IBM Corp. Released 2013; Armonk, NY, USA). The structures of the left and right frontal cortices were investigated separately. The associations between PIUQ scores and volumetry were analyzed with multiple linear regressions. We created separate models for each structure as a dependent variable and PIUQ scores as independent variables. Head size correction was carried out by entering intracranial volume as additional independent variable (Perlaki et al., 2014). The assumptions of the analysis (Chan, 2004) were satisfied. To reduce type-I error, multiple comparisons were corrected using the Benjamini–Hochberg method (false discovery rate = 0.05; Benjamini & Hochberg, 1995). Data of males and females were analyzed separately. Sex differences in PIUQ scores were tested with independent samples *t*-tests.

### Ethics

The research was approved by the local ethical review committee and was carried out in accordance with the Code of Ethics of the World Medical Association (Declaration of Helsinki), and informed consent was obtained from all of the participants involved in the study.

## Results

### Descriptive data

The mean score for the PIUQ was 31.05 (*SD* = 9.41, range: 18–53). The subscales mean scores were 9.04 (*SD* = 3.08, range: 6–17) for Obsession, 11.09 (*SD* = 3.74, range: 6–24) for Neglect, and 10.91 (*SD* = 3.96, range: 6–20) for Control Disorder. Significant difference was found between males and females in the Neglect subscale [*t*(142) = 2.284, *p* < .05] such that males (mean scores = 11.88, *SD* = 4.26) scored higher than females (mean scores = 10.46, *SD* = 3.14).

### Volumetry

In males, Control Disorder was positively associated with the volumes of left lateral orbitofrontal and right precentral gyri, whereas Neglect positively correlated with volumes of bilateral orbitofrontal gyri and right precentral gyri. The PIUQ total score was also positively related to the left lateral orbitofrontal gyrus volume. No correlation was found between Obsession and the frontal lobe structures. However, none of these correlations survived correction for multiple comparisons.

In females, Control Disorder was positively associated with the volumes of left precentral gyrus, right POp, and PTr. Obsession was positively correlated with the bilateral POp. Neglect was positively associated with left precentral gyrus, right POp, and PTr volumes, whereas the PIUQ total score was correlated with the right POp and PTr volumes. Only the correlations of the Obsession and Neglect scales with the volume of right POp survived the correction for multiple comparisons. The results of the multiple linear regression analyses are shown in Table 1.

**Table 1.** The associations between the subscales and the total score of PIUQ and the volumes of frontal lobe structures in males and females, separately

			Control Disorder		Obsession		Neglect		Total score	
			Male	Female	Male	Female	Male	Female	Male	Female
Caudal middle frontal gyrus	Left	*t*	1.361	1.517	−0.376	0.371	1.363	1.109	1.006	1.178
		β	0.188	0.182	−0.052	0.045	0.187	0.135	0.139	0.143
	Right	*t*	0.553	0.924	−1.304	0.222	0.344	0.688	−0.052	0.722
		β	0.081	0.110	−0.186	0.027	0.050	0.082	−0.008	0.086
Lateral orbitofrontal gyrus	Left	*t*	2.053*	0.030	1.348	0.321	2.230*	−0.106	2.251*	0.082
		β	0.286	0.004	0.189	0.038	0.307	−0.012	0.310	0.010
	Right	*t*	1.436	−0.131	1.301	0.361	2.179*	−0.097	1.960	0.027
		β	0.213	−0.015	0.191	0.042	0.314	−0.011	0.284	0.003
Medial orbitofrontal gyrus	Left	*t*	−0.058	0.776	−0.129	0.859	1.531	1.164	0.593	1.033
		β	−0.009	0.090	−0.019	0.100	0.221	0.135	0.087	0.120
	Right	*t*	1.749	0.412	0.351	0.078	1.219	−0.073	1.340	0.183
		β	0.262	0.050	0.053	0.009	0.184	−0.009	0.201	0.022
Pars opercularis	Left	*t*	−0.134	0.982	−1.582	2.227*	−1.282	1.101	−1.126	1.547
		β	−0.019	0.112	−0.213	0.250	−0.175	0.126	−0.154	0.176
	Right	*t*	−0.430	2.723*	0.669	3.052^a^^,^*	0.010	2.978^a^^,^*	0.052	3.293*
		β	−0.067	0.297	0.103	0.331	0.002	0.323	0.008	0.353
Pars orbitalis	Left	*t*	0.542	1.168	0.246	1.120	1.555	1.142	0.970	1.287
		β	0.073	0.133	0.033	0.128	0.205	0.130	0.129	0.146
	Right	*t*	−0.602	0.814	0.106	1.432	0.058	0.079	−0.179	0.856
		β	−0.078	0.093	0.014	0.162	0.008	0.009	−0.023	0.098
Pars triangularis	Left	*t*	0.362	0.168	0.224	1.403	0.162	0.441	0.288	0.686
		β	0.049	0.019	0.030	0.161	0.022	0.051	0.039	0.079
	Right	*t*	−0.505	2.524*	0.292	1.541	0.308	2.004*	0.029	2.333*
		β	−0.068	0.291	0.039	0.183	0.041	0.235	0.004	0.272
Precentral gyrus	Left	*t*	1.298	2.369*	1.507	0.125	1.648	2.501*	1.742	1.938
		β	0.211	0.281	0.241	0.015	0.264	0.297	0.278	0.233
	Right	*t*	2.242*	1.477	0.030	0.405	2.412*	1.010	1.931	1.138
		β	0.317	0.180	0.004	0.050	0.336	0.124	0.274	0.140
Rostal middle frontal gyrus	Left	*t*	0.268	0.770	−0.897	−0.096	1.589	0.704	0.496	0.554
		β	0.040	0.094	−0.132	−0.012	0.232	0.086	0.074	0.068
	Right	*t*	1.693	−0.375	1.068	0.104	1.222	0.143	1.563	−0.082
		β	0.254	−0.046	0.160	0.013	0.184	0.018	0.234	−0.010
Superior frontal gyrus	Left	*t*	1.335	1.816	−0.107	0.625	0.828	1.498	0.853	1.531
		β	0.246	0.215	−0.020	0.076	0.153	0.179	0.158	0.183
	Right	*t*	0.864	1.544	−1.309	0.508	−0.119	1.383	−0.134	1.332
		β	0.145	0.193	−0.127	0.064	−0.020	0.174	−0.023	0.167

*Note.* PIUQ: Problematic Internet Use Questionnaire.

a Significant associations after Benjamini–Hochberg correction.

* *p* < .05.

We measured gender differences in the correlation between PIUQ scales and right POp, where we found significant associations only in females. We found significant interactions between gender and PIUQ scales [Obsession subscale: *F*(2, 141) = 12.883, *p* < .01; Neglect subscale: *F*(2, 141) = 11.783, *p* < .01].

### VBM

**Figure 1.**
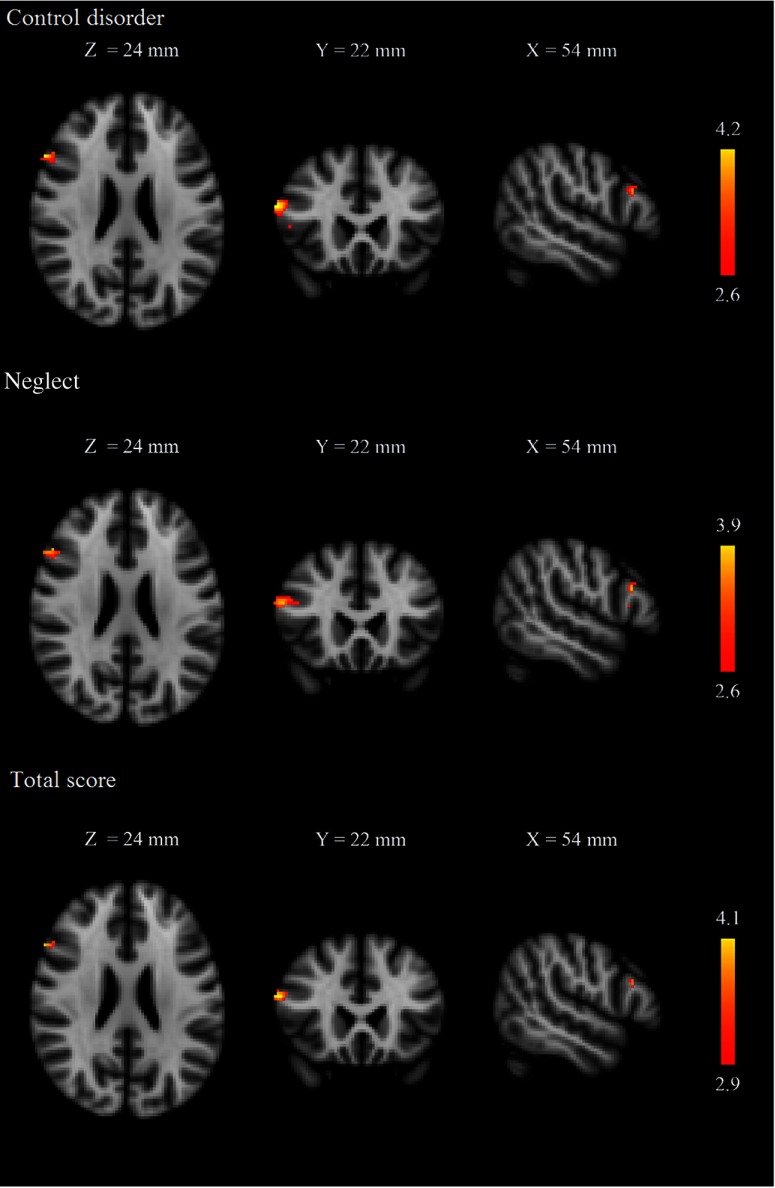
Voxelwise analysis of the frontal cortex. Red–yellow indicate voxels demonstrating significant positive correlation between the amount of gray matter mass in pars opercularis and PIUQ scores in females. Color bar represents *z* values. The background image is the MNI152 standard space T1 template. *X*, *Y*, and *Z* values indicate the MNI slice coordinates in millimeter. Images are shown in radiological convention

The subscales of PIUQ were used separately to predict the amount of gray matter of brain areas in the frontal lobe. Positive correlations were found between the amount of gray matter mass in right POp and Control Disorder, Neglect, and total score in females (Figure 1). PIUQ scores showed no significant association with gray matter mass of the frontal lobe in males.

## Discussion

In this study, we measured the structural neural correlates of IA in healthy young male and female habitual Internet users. We found a positive relationship between the PIUQ scales and the volume of the right POp in females using two different brain analysis techniques (VBM and FreeSurfer). Previous studies showed negative correlation between IA and volumes of the frontal cortex; however, these studies used ROIs related to control functions (Kühn & Gallinat, 2015; Yuan et al., 2011) and brain’s reward system (Altbäcker et al., 2016). These papers suggest that the decreased volume of the frontal cortex is associated with the common features of addictions, such as self-control deficit, social problems, impulsivity, and craving (Kuss et al., 2014; Van Rooij & Prause, 2014). Our primary aim was to investigate IA-related gender differences in the frontal cortex. According to previous studies, males are absorbed in online gaming, whereas females use Internet merely for communication (Kimbrough et al., 2013; Weiser, 2000). Thus, compared to males, females do not neglect their social relationships. This was supported by our finding that females reached lower scores on the Neglect subscale of the PIUQ than males. Due to the deficit in control functions, a negative correlation can be described between the IA and the frontal regions. On the contrary, if the social communication is not neglected, a positive correlation can be hypothesized between the volume of social interaction-related structures and the IA. The POp plays an important role in the success of social interactions as well as the control functions (Nishitani, Schürmann, Amunts, & Hari, 2005). The positive relationship between the PIUQ score and the POp could be the results of the increased use of this area during frequent communication.

The absence of the positive association between the IA and IFC in males might be due to the gender differences in Internet using habits. That is, males tend to use the Internet for entertainment purposes, for example, online gaming (Ko et al., 2005); whereas females are more likely to use the Internet for communication, education, and social media activity (Kimbrough et al., 2013; Weiser, 2000). A longitudinal study (Van Deursen, Van Dijk, & Ten Klooster, 2015) showed that sex differences in Internet using habits are consistent over years; however, the number of females in online gaming shows a growing tendency. Provided that females use the Internet to communicate with others, the positive correlation is reasonable between PIUQ scores and the size of IFC. Although the Broca area is on the left hemisphere, the right-sided homologue area, the right POp, can affect the social interactions too, for instance to make sense of emotional expression (Nishitani et al., 2005). The gray matter volume of the right POp showed negative correlation with severe social communication problems; hence, the increased volume may refer to better interpersonal skills (Yamasaki et al., 2010).

Limitations of this study include not measuring the Internet-using habits directly. However, the aforementioned difference might be visible in males scoring higher than females on the Neglect subscale that refers to neglecting social life due to Internet use. Furthermore, although we measured the association between IA and gray matter volume of frontal lobe regions, a longitudinal study design is needed in order to examine a causal relationship. With cross-sectional studies only correlations could be studied, the causality in IA-related brain–behavior relationship cannot be investigated. To decide, if the increased frontal volume results in problematic Internet use or the IA is the reason of specific structural features of brain, longitudinal studies are needed.

## Conclusions

In sum, we suggest that the association between IFC and IA could be caused by the effort of controlling the impulsive behavior and the frequency of social interaction. Hence, the positive correlation between PIUQ and the volume of POp was only found in females.
